# Intolerance of Uncertainty and Anxiety-Related Dispositions Predict Pain During Upper Endoscopy

**DOI:** 10.3389/fpsyg.2019.01112

**Published:** 2019-05-15

**Authors:** Marco Lauriola, Manuela Tomai, Rossella Palma, Gaia La Spina, Anastasia Foglia, Cristina Panetta, Marilena Raniolo, Stefano Pontone

**Affiliations:** ^1^Department of Social and Developmental Psychology, Faculty of Medicine and Psychology, Sapienza University of Rome, Rome, Italy; ^2^Department of Dynamic and Clinical Psychology, Faculty of Medicine and Psychology, Sapienza University of Rome, Rome, Italy; ^3^Department of Surgical Sciences, Faculty of Medicine and Dentistry, Sapienza University of Rome, Rome, Italy

**Keywords:** intolerance of uncertainty, anxiety-sensitivity, procedural anxiety, pain catastrophizing, esophagogastroduodenoscopy, prospective-study

## Abstract

Although sedatives can defuse anxiety and relieve pain, Esophagogastroduodenoscopy (EGD) still is uncomfortable and threatening for some patients. Identifying patients who tolerate digestive endoscopy less well remains difficult. Using a prospective design and a multimodal assessment of pain, the present study evaluated how anxiety-related variables predicted subsequent pain outcomes. Sixty-two consecutive patients referred for elective EGD were assessed for intolerance of uncertainty (IU), procedure-related worries, anxiety sensitivity and health distress before endoscopy. During endoscopy, a doctor rated patients’ pain behavior. After complete recovery from sedation, the patients retrospectively rated endoscopy pain and situation specific catastrophizing thoughts. Descriptive analyses showed that patients undergoing EGD for the first time were more distressed and anxious than patients accustomed to the procedure and needed a higher sedative dose. Notwithstanding sedation, the behavioral rating of pain was above the cut-off value for probable pain for more than half of the patients. IU assessed before endoscopy predicted situational pain catastrophizing (PC) and self-reported pain after endoscopy through procedure related worries. Situational PC not only mediated the effect of worry, but also female gender and younger age were associated with self-reported pain through increased catastrophizing thoughts. Health distress and anxiety sensitivity predicted PC only for women, younger patients, and those not accustomed to the procedure. Our study showed that psychological preparation before sedation is needed especially for first-timers, women, and younger patients, addressing maladaptive cognitive beliefs and acquainting patients with the somatic sensations that they might experience during the procedure.

## Introduction

Esophagogastroduodenoscopy (EGD) is a diagnostic procedure carried out using a flexible probe equipped with a camera, which allows the mucous membrane of the esophagus, stomach, and duodenum to be explored visually. The examination lasts a few minutes, is safe, and has many benefits, such as accurate diagnosis and guidance on effective interventions for upper gastrointestinal conditions. Although EGD is well tolerated, the patients may experience mild to moderate discomfort, and the prospect of inserting the probe through the oral cavity, then sliding it into the stomach, may evoke fears such as that of unpleasant physical sensations, adverse diagnostic outcomes (e.g., cancer), and insufficient sedation (Brandt, 2001). Because of these concerns, the most anxious patients become distressed to the point of preventing EGD from being performed or continued (e.g., Trevisani et al., 2004; El-Hassan et al., 2009; Mitsonis et al., 2011). Moreover, procedural anxiety prevents adherence to diagnostic screening tests, becoming a barrier to the early diagnosis of cancer and other severe chronic conditions (Oikonomidou et al., 2011; Trevisani et al., 2014).

Conscious sedation reduces patient anxiety and discomfort (e.g., Mui et al., 2005) but is not exempt from medical complications, needs additional time and specialized personnel to prepare the patients and monitoring their recovery, and the patients themselves must refrain from activities such as driving and working for hours after EGD. Noteworthy, highly anxious patients are more difficult to sedate and require higher doses to maintain an acceptable level of sedation (e.g., Lee et al., 2004; Bal et al., 2012; Gürbulak et al., 2018). Psychological preparation for EGD is an effective non-pharmacological intervention to defuse pre-procedural anxiety before sedation (Maguire et al., 2004; García Sierra et al., 2013; Kowsalya et al., 2015; Behrouzian et al., 2017; Liu et al., 2018; Ghonaem and Ibrahim, 2019) but can be time-consuming and may cause a delay in the flow of patients, especially if performed routinely the same day of endoscopy (e.g., Behrouzian et al., 2017).

There is a need to prioritize patients who are at greater risk of experiencing clinically relevant anxiety, targeting those to be psychologically prepared according to their needs and personality characteristics. Female gender, younger age, and no previous endoscopy experience are known risk factors for pre-procedural anxiety and low EGD tolerability (Davies and Roy, 2013; Lee et al., 2014; Gürbulak et al., 2018; Sayilan and Oztekin, 2018). However, the psychological characteristics that make these groups more difficult to examine are still unclear, and identifying which patients might tolerate digestive endoscopy less well remains challenging (Hazeldine et al., 2010; Bal et al., 2012). Being almost exclusively focused on anxiety symptoms, previous research has overlooked the role of cognitive characteristics underlying these symptoms (Jones et al., 2004; Essink-Bot et al., 2007; Pontone et al., 2015; Behrouzian et al., 2017). Three of these, IU, anxiety sensitivity, and pain catastrophizing (PC) are worthy of attention.

Intolerance of uncertainty (IU) is a cognitive disposition involved in the emergence and maintenance of anxiety disorders and depression, influencing how people react to uncertain events, however unlikely, appraising them as unfair, unacceptable, and threatening (Carleton et al., 2007a). Worrying is the most direct consequence of IU (Dugas et al., 2005; Shihata et al., 2016). People high on IU tend to engage in a mental simulation about what may or may not occur, erroneously believing that anticipating the feared events might help them to be prepared in case things go awry (De Bruin et al., 2007; Newman et al., 2013). Unfortunately, these thoughts do not entail effective coping strategies, and the feelings of anxiety may persist or even be boosted by worrying rumination (Heiden and Broeke, 2009).

According to Carleton (2016), IU reflects a more basic fear of the unknown “caused by the perceived *absence of information at any level of consciousness*” (p. 5, italics added). This definition is consistent with evidence showing that “first-timers,” lacking experiential information about the procedure, tend to feel greater anxiety than “repeaters” (Davies and Roy, 2013) and with studies supporting the effectiveness of psychological preparation, in which the patients are acquainted with the physical sensations that may arise during EGD (Maguire et al., 2004; García Sierra et al., 2013; Behrouzian et al., 2017; Liu et al., 2018; Ghonaem and Ibrahim, 2019).

The fear of pain is among the most prominent causes of anxiety for EGD patients (Brandt, 2001). Different from IU, anxiety sensitivity represents the “fear of arousal-related sensations” that everyone experience in anxiety-inducing situations (Taylor et al., 2007). Although the definition of anxiety sensitivity has recently broadened to include the fear of psychological and social consequences of anxiety (Ebesutani et al., 2013), the construct remains more narrow in scope than IU. This argument prompted Carleton et al. (2007b) to suggest that anxiety sensitivity is likely dependent on IU, which maintains the status of broad transdiagnostic vulnerability factor for a wide range of affective disorders.

Anxiety sensitivity is instead a significant factor for panic disorders and hypochondria (Norton et al., 2005), engendering catastrophic misinterpretations (e.g., a heart attack) of harmless physical sensations (e.g., shallow breathing, palpitations) associated with hyperarousal (Ohst and Tuschen-Caffier, 2018). According to this definition, EGD patients high on anxiety sensitivity, especially those not accustomed with the procedure, might misinterpret the somatic sensations caused by the endoscope as signs of an imminent danger (e.g., chocking, having an iatrogenic perforation), panicking about what was going to happen. Moreover, previous research has also shown that anxiety and anxiety sensitivity were reliably associated with a heightened experience of acute and chronic pain (e.g., Ocañez et al., 2010; Catalano et al., 2017).

Pain catastrophizing (PC) is a third cognitive construct that might be involved in the relationship between procedural anxiety and EGD tolerability. PC is a mindset of exaggerated negative cognitions and emotional schemas that describe people’s beliefs, appraisals and feelings related to actual or expected pain experience (Quartana et al., 2009). As a multidimensional construct, PC encompasses ruminative thoughts about pain and failure to defuse them, perceived inability to cope with painful situations, and amplification of pain or fear of the negative consequences of pain (Sullivan et al., 1995). These characteristics resonate those of anxiety sensitivity, especially regarding the magnification of potentially harmful stimuli. For instance, Stewart and Asmundson (2006) maintained that patients high on anxiety sensitivity might be more apt to make catastrophic thoughts about pain when confronted with noxious stimuli, a claim supported by several empirical studies (for a review see Olthuis and Asmundson, 2019).

Similar to IU and anxiety sensitivity, PC has long been considered a dispositional trait involved in the maintenance of chronic pain and disability (Quartana et al., 2009). However, research has shown that PC has the characteristics of a situational variable, yielding more robust correlations with pain outcomes than dispositional PC for acute pain and experimentally induced stimulation (Strulov et al., 2007; Campbell et al., 2010; Grosen et al., 2016). Because an EGD is more alike to a “clinical experiment” than to a chronic condition, we believe that situational PC has a greater potential to reveal sound relationships with EGD tolerability than dispositional PC.

**FIGURE 1:**
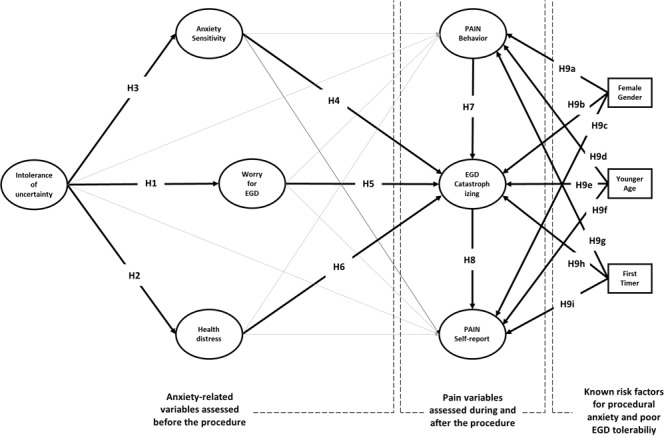
Theoretical model and hypotheses.

How IU, anxiety sensitivity and PC shape patient’s experience of EGD? Previous research has overlooked the role of these cognitive characteristics. Moreover, no single study has assessed IU, anxiety sensitivity and PC in EGD patients, nor has examined the predictive role of psychological variables using a two-stage prospective design. As shown in Figure 1, we assessed IU, anxiety sensitivity, procedure-related worry and health distress before the procedure, observed pain behavior during the procedure, and collected pain perceptions and situation-specific catastrophizing thoughts after the procedure (for details see section Materials and Methods).

Because IU is known to lead to excessive preoccupation, we hypothesized a direct relationship of this variable with EGD worries (H1). According to the transdiagnostic hypothesis, we also expected IU to be linked with patient’s health-distress (H2), operatively defined as a combination of depression and anxiety feelings before the procedure. Because IU entails a more general fear of the unknown than anxiety sensitivity, we also hypothesized that the greater the fear of the unknown, the greater the fear of the unknown consequences of procedure-related anxiety (H3). According to H4–H6, we expect that the set of cognitive variables can predict situation-specific catastrophizing thoughts reported by the patients after the procedure. PC has a central role in our model. We expect situation specific catastrophizing thoughts to magnify the experience of pain during the EGD (H7) and lead to greater pain reporting after EGD (H8).

The model also considers female gender, younger age, and no previous endoscopy experience as external sources that can predict the intensity of pain and PC (H9a-i). Adding these variables to the model has several advantages. First, it allows for controlling the effects of known risk factors for pre-procedural anxiety and poor EGD tolerability. Second, it allows studying their moderation effects on the relationships between psychological and pain variables. Moderation effects in a predictive model test “under which circumstances” or “for whom,” an independent variable is more (or less) strongly associated with the outcomes. For instance, if the moderation effect of gender on the relationship between anxiety sensitivity and PC turns out statistically significant, that entails anxiety sensitivity to be differently predictive of PC for men and women.

## Materials and Methods

### Participants

Inclusion criteria for the study were age over 18 years and knowledge of the Italian language. Exclusion criteria were a history of psychiatric disorders, use of antidepressants, adrenoreceptor antagonists, or opioids, current or recent chronic pain syndrome. Eighty consecutive outpatients referred for EGD at the Endoscopy Unit of “Sapienza” University of Rome were eligible for the present study. Seven patients refused to participate (9%). Five (6%) and two (3%) patients did not complete the psychological scales before endoscopy and refused to answer pain questions after endoscopy, respectively. Four patients (5%) were excluded because of a history of psychiatric disorders or current use of medications.

The analyses were carried out on 62 patients (31 women and 31 men), 35 (54%) of which had previous EGD experience. The mean age of the sample ranged from 25 to 86 years (*M* = 58.24; *SD* = 14.88). Of the 62 patients referred for EGD, only five patients had signs of serious diseases (e.g., unexplained weight loss, anemia, and fecal occult blood). The remaining 57 patients were either symptomatic (*n* = 43; e.g., epigastric pain, dyspepsia) or follow-up after surgery (*n* = 14). The EGD lasted on average 7.43 min (*SD* = 5.38) and were performed under conscious sedation using a standard endoscope. Patients received a dose of 2–5 mg of midazolam, with a dosage protocol of 0.07 mg/kg. The BMI ranged from 16 to 35 kg/m^2^ (*M* = 24.26; *SD* = 3.71). Considering the BMI normal range 18–25, one patient was underweight, 24 were overweight, and five were obese (i.e., BMI > 30). The final endoscopic diagnosis was categorized as inflammation (*n* = 30; i.e., anthropathy, duodenitis, esophagitis), lesion (*n* = 12; i.e., gastric erosion, polyposis, ulcer, esophageal varicose veins), other conditions (*n* = 16; e.g., hiatal hernia, Barrett’s esophagus), or negative endoscopy (*n* = 3). Because of an incomplete EGD, the diagnosis was unknown for one patient.

### Procedure

Patients were recruited at only one university clinic. Upon arrival, a specializing doctor and a psychologist invited eligible patients to participate in the study. After receiving informed consent from the patient, the psychologist took the patient to a comfortable room and gave him a confidential questionnaire including measures of trait anxiety and depression, IU, anxiety sensitivity, and worry (see instruments). The psychologist was in the room and assisted the patient upon request. A progressive ticket number was assigned to each patient that had to be delivered to the endoscopist doctor in the operating room. Before endoscopy, the doctor verified the absence of exclusion criteria for the study, transcribed patient’s anamnestic data, and then proceeded to the EGD. Only one endoscopist was involved in the study and performed all the exams. During endoscopy, a specializing doctor observed the patients and rated patient’s pain and sedation level. The specializing and the endoscopist doctors were blinded to the patient’s answers to the psychological scales administered before endoscopy. After complete recovery from sedation, the endoscopist doctor invited the patient to answer questions about pain and situational PC, which were placed with the anamnestic data into a sealed envelope on which the patient’s progressive number was transcribed. This study was carried out in accordance with the recommendations of the Code of Ethics for Research in Psychology, Italian Association of Psychology. The protocol was approved by the Ethical Committee of the Department of Dynamic and Clinical Psychology, “Sapienza” University of Rome. All subjects gave written informed consent in accordance with the Declaration of Helsinki.

### Variables and Instruments

#### Health Distress

The Hospital Anxiety and Depression Scale (HADS) includes seven items for anxiety and seven for depression symptoms, each rated on a 4-point severity scale. The total score is a valid measure of health distress (Snaith, 2003). In the present study, we used the Italian validated version (Iani et al., 2014). The Cronbach’s alpha coefficients were 0.74, 0.60, and 0.75 for anxiety, depression, and the total score, respectively. There is no cut-off for the total score, but a subscale score greater than seven is commonly used for fast screening of medical patients at risk for health anxiety and depression (Stern, 2014).

#### Intolerance of Uncertainty

The Italian version of the Intolerance of Uncertainty Inventory (IUI-A) is a 10-item scale developed to assess the tendency for a person to consider uncertainties in life to be unacceptable and threatening (Carleton et al., 2010). The items were administered using a 5-point scale (1 = “not at all characteristic of me,” 5 = “entirely characteristic of me”). The total score is a valid measure of IU as currently defined (α = 0.94, in this study). The normal range for an Italian community sample is between 9 and 46 (Lauriola et al., 2018).

#### Anxiety Sensitivity

The ASI-3 Italian version (Ghisi et al., 2016) is an 18-item scale assessing tendency to fear the symptoms of anxiety. Responses are given using a five-point Likert scale. The ASI-3 provides three subscale scores: fear of somatic sensations, fear of loss of cognitive or psychological control, and fear of publicly observable anxiety symptoms. In the present study, we administered only the somatic and cognitive subscales (Cronbach’s alpha coefficients were 0.89 and 0.90, respectively). To our knowledge, normal ranges for the subscales are currently unpublished.

#### Worry Questionnaire

To capture cognitive activity associated with pre-procedural anxiety, we developed a Worry for Medical Procedures Scale (WMPS) by rewording the items of the Penn-State Worry Questionnaire (Hopko et al., 2003) in the context of invasive diagnostic examinations. The WMPS included eight items, four of which described specific concerns about the procedure. Two items described worries about test results, while two items described worries about general health (Cronbach’s alpha for the total score was 0.90).

#### Behavioral Rating of Pain

Because the patient was unable to communicate during EGD, we rated the pain responses during endoscopy using the Pain Assessment in Advanced Dementia Scale (PAINAD; Warden et al., 2003). A medical doctor in the operating room assessed the patient’s breathing, negative vocalizations, facial expression, body language, and consolability. A total score was obtained from 0 to 10 with higher scores indicating more severe pain (Cronbach’s alpha for the total score was 0.90). A score above two indicates possible pain, while a score above four indicates moderate pain (Zwakhalen et al., 2012).

#### Self-Assessment of Pain

Pain intensity was assessed using visual-analog, numeric, verbal, and face scales. The Visual Analog Scale (VAS) required the patient to place a mark on a 10 cm long horizontal segment that went from “no pain” to “worst imaginable pain.” There were no words on the segment between the two ends. We obtained a continuous score in centimeters, ranging from 0 to 10. The numeric scale asked the patient to evaluate how painful was the procedure using integer numbers from 0 to 10, with higher numbers indicating lower pain intensity. The numeric response scale was intentionally reversed to prevent carryover effect. The score was reversed before the analysis. The verbal scale included five verbal descriptors placed in a ranked order. Very severe, severe, moderate, mild, very mild, and no pain was coded using numbers from 5 to 0, respectively. Last, patients were asked to report the experienced pain selecting from six drawings of facial expressions of pain.

#### Situational Pain Catastrophizing

To capture catastrophizing thoughts occurring during EGD, we used the same items included in the Italian version of the dispositional PC Scale (Monticone et al., 2012), changing the instructions and rewording the items in the past tense. The patients were asked to refer to thoughts, feelings, and physical sensations experienced during the procedure. Paralleling the standard PCS, we got a total score for situational pain-catastrophizing (13 items, Cronbach’s alpha = 0.93) and three subscale scores for pain-helplessness (6 items, Cronbach’s alpha = 0.88), pain-rumination (4 items, Cronbach’s alpha = 0.89), and pain-magnification, 3 items, Cronbach’s alpha = 0.63).

### Data Analysis

We performed a partial least squares structural equation modeling analysis (PLS-SEM) using Smart PLS 3 (Ringle et al., 2015). PLS-SEM is a non-parametric path analysis method recommended when the goal of the study is prediction rather than theory testing, and the sample size does not permit using standard SEM (Hair et al., 2017). PLS-SEM makes no assumptions regarding the underlying distribution of the variables, working well with non-normal or highly skewed data (Hair et al., 2017).

The model evaluation comprises two stages: the assessment of the “measurement model,” dealing with the relationships between the empirical indicators and the latent variables, and the evaluation of the “structural model”, which represents the direct and indirect relationships between latent variables. Four quality criteria determine the adequacy of the measurement model. First, all indicators variables should load on the corresponding latent variables above 0.50 (indicator reliability). Second, the Composite Reliability (CR) of each latent variable should be at least above 0.60, or preferably above 0.70 (construct reliability). Third, the Average Variance Extracted (AVE), measuring the proportion of variance in the indicators that is accounted for by the corresponding latent variable, should be 0.50 or higher (convergent validity). Last, the square roots of the AVE for each latent variable should be larger than the estimated correlations of that latent variable with other variables in the model (discriminant validity).

The evaluation of the structural model is based on how well the model predicted the endogenous variables. First, we examined the determination coefficients (*R*^2^) for the endogenous latent variables. According to Hair et al. (2017), *R*^2^-values of 0.75, 0.50, and 0.25 represent high, moderate, and low thresholds, respectively. The predictive accuracy of the model is also evaluated in terms of cross-validation. For this purpose, a *Q*^2^ cross-validation index is obtained for each endogenous variable using a blindfolding procedure assessing the ability of the model to predict omitted data not used for estimation (Hair et al., 2017). Positive *Q*^2^-values indicate that the model has predictive relevance. The higher is the *Q*^2^, the higher the predictive accuracy of the model. The significance of the direct path coefficients is tested using non-parametric confidence intervals obtained from 5000 bootstrap resampling iterations (Streukens and Leroi-Werelds, 2016). Although the two-tailed test type remains the default option in PLS-SEM, a one-tailed test is suitable for small sample analyses and theoretically sound directional hypotheses (Kock, 2015). Besides evaluating the significance of the path coefficients, it is advised to assess their effect size using the *f*^2^, which is the change in *R*^2^ in an endogenous variable when a specific path is omitted from the model. Following Hair et al. (2017), 0.02, 0.15, and 0.35 represent small, medium, and large effect sizes, respectively.

We hypothesized and specifically tested moderating relationships involving age, gender, and EGD experience with anxiety sensitivity, worry, and health distress on situational PC, self-reported pain, and pain behavior. These relationships were tested adding specific interaction terms to the PLS model depicted in Figure 1. Each interaction was obtained as the product of the latent variable score for the predictor (e.g., anxiety sensitivity) times the moderator (e.g., gender) after mean centering both factors. Before running the analyses, we checked for multicollinearity among moderators, a violation of regression assumptions occurring when the interaction terms in the model are such correlated to provide redundant information about the dependent variable. The Variance Inflation Factor (VIF) for each moderator is commonly used to assess multicollinearity problems. Under ideal conditions, the VIF should be less than 3, with VIF values less than 4 (or more leniently 5) deemed acceptable (Garson, 2016). With all 27 product terms in the model, twenty showed a VIF < 3, six had a VIF < 4, and only one was 4.1. Multicollinearity did not appear to be a severe problem in the analyses.

## Results

Table 1 reports descriptive statistics for sedation and pain-related variables assessed in the total sample and broken down by gender and endoscopy experience. Regarding sedation, the Ramsey score was between 2 and 3 for 85% of the patients, showing that most of them were awake, cooperative, and responsive to commands during the EGD. To attain an adequate level of sedation the patients needed an average dose of 2.97 mg of Midazolam. However, the patients undergoing EGD for the first time needed a higher dose than patients accustomed to the procedure, while the dose administered to women and men was the same. Because there were no between-group differences in BMI, the administration of a higher dose to first-timers was not due to differences in the body mass of the patients.

**Table 1:** Sedation and pain variables in the total sample and broken by gender and previous EGD experience.

	Total sample		Men		Women				First-timers		Experienced	
Variables (range)	*N* = 62		*N* = 31		*N* = 31				*N* = 27		*N* = 35			
	*M*	*DS*	*M*	*DS*	*M*	*DS*	*t* (61)	*p*	*M*	*DS*	*M*	*DS*	*t* (61)	*p*
Ramsey sedation score (1–5)	2.84	(0.75)	2.77	(0.76)	2.90	(0.75)	0.67		2.93	(0.68)	2.77	(0.81)	0.80	
Midazolam (mg/l)	2.97	(1.16)	2.84	(1.13)	3.10	(1.19)	0.88		3.41	(0.97)	2.63	(1.19)	2.76	^∗∗^
BMI (Kg/m^2^)	24.28	(3.90)	24.61	(3.37)	23.95	(4.40)	0.67		24.67	(3.90)	23.98	(3.93)	0.69	
PAINAD total (0–13)	1.68	(2.06)	1.48	(2.26)	1.87	(1.86)	0.74		1.89	(2.03)	1.51	(2.11)	0.71	
Breathing (0–2)	0.13	(0.34)	0.12	(0.34)	0.13	(0.33)	0.00		0.15	(0.36)	0.11	(0.32)	0.39	
Negative vocalizations (0–2)	0.34	(0.51)	0.32	(0.54)	0.35	(0.49)	0.25		0.41	(0.50)	0.29	(0.52)	0.93	
Facial expression (0–2)	0.44	(0.53)	0.35	(0.55)	0.52	(0.51)	1.20		0.37	(0.49)	0.49	(0.56)	0.84	
Body language (0–2)	0.44	(0.53)	0.39	(0.56)	0.48	(0.51)	0.71		0.52	(0.51)	0.37	(0.55)	1.08	
Consolability (0–2)	0.34	(0.51)	0.29	(0.53)	0.39	(0.50)	0.74		0.44	(0.51)	0.26	(0.51)	1.45	
Self-report pain total (z-score)	0.00	(1.00)	–0.31	(0.91)	0.31	(1.00)	2.59	^∗^	0.14	(0.95)	–0.11	(1.04)	0.98	
Verbal scale (0–5)	4.09	(3.99)	2.91	(3.03)	5.26	(4.52)	2.41	^∗^	4.30	(4.40)	3.92	(3.71)	0.37	
Visual analog scale (0–100)	1.61	(1.38)	1.26	(1.29)	1.97	(1.40)	2.07	^∗^	1.85	(1.32)	1.43	(1.42)	1.20	
Face scale (0–5)	2.21	(1.16)	1.81	(0.95)	2.61	(1.23)	2.89	^∗∗^	2.59	(1.15)	1.91	(1.09)	2.36	^∗^
Numeric scale (0–10)	2.48	(2.60)	2.03	(2.66)	2.94	(2.49)	1.38		2.33	(2.29)	2.60	(2.84)	0.40	
Pain catastrophizing (0–36)	8.20	(9.03)	4.93	(6.33)	11.35	(10.17)	2.95	^∗∗^	10.67	(9.60)	6.24	(8.17)	1.95	^†^
Pain helplessness (0–13)	0.48	(0.66)	0.22	(0.48)	0.73	(0.72)	3.22	^∗∗^	0.63	(0.71)	0.35	(0.61)	1.64	
Pain rumination (0–17)	0.87	(0.93)	0.51	(0.66)	1.21	(1.03)	3.11	^∗∗^	1.13	(1.00)	0.66	(0.83)	1.99	^†^
Pain magnification (0–8)	0.51	(0.82)	0.53	(0.69)	0.48	(0.94)	0.23		0.63	(0.98)	0.41	(0.67)	1.03	

*^∗∗∗^p < 0.001; ^∗∗^p < 0.01; ^∗^p < 0.05; ^†^p < 0.05 (one-tailed test).*

Notwithstanding sedation, the behavioral rating of pain score was above the cut-off value for probable pain for 29 patients (53%). No differences were found by gender and EGD experience (Table 1). However, the data suggested sizeable individual differences in pain behavior, especially for first-timers and women. After the EGD, women reported more pain than man. First-timers also reported more pain than repeaters. However, the statistical tests attained significance only for the face pain scale. When asked to disclose situation-specific catastrophizing thoughts, women reported more helplessness, rumination, and general catastrophizing scores than men. Similarly, first-timers reported more rumination and general catastrophizing scores than men than experienced patients (*p* < 0.05, one-tailed).

Table 2 reports descriptive statistics for the psychological variables assessed before endoscopy. The IU score was in the normal range and there was no difference by gender or endoscopy experience. Regarding anxiety sensitivity, women obtained significantly higher scores than men. First-timers were significantly more distressed and anxious than patients with previous EGD experience (*ps* < 0.05, one-tailed). Similarly, first-timers referred to be more worried than experienced patients, regarding the procedure and its clinical outcomes (*ps* < 0.05, one-tailed). Women also reported more concerns about the procedure than men (*ps* < 0.05, one-tailed).

**Table 2:** Psychological variables in the total sample and broken by gender and first-time EGD.

	Total sample		Men		Women				First-timers		Experienced	
Variables (range)	*N* = 62		*N* = 31		*N* = 31				*N* = 27		*N* = 35			
	*M*	*DS*	*M*	*DS*	*M*	*DS*	*t* (61)	*p*	*M*	*DS*	*M*	*DS*	*t* (61)	*p*
IU total (10–50)	23.25	(9.64)	22.86	(10.1)	23.63	(9.36)	0.30		24.96	(7.91)	21.81	(10.80)	1.26	
AS total (0–48)	11.93	(9.90)	9.24	(6.76)	14.53	(11.7)	2.11	^∗^	13.44	(9.07)	10.66	(10.51)	1.08	
AS cognitive (0–24)	5.44	(5.29)	3.97	(3.66)	6.87	(6.18)	2.17	^∗^	5.77	(4.62)	5.16	(5.86)	0.45	
AS sens. physical (0–24)	6.01	(6.49)	5.28	(4.21)	7.67	(6.01)	1.76	^†^	7.67	(5.23)	5.50	(5.23)	1.58	
Health distress (1–28)	11.86	(5.56)	10.90	(5.83)	12.83	(5.19)	1.33		13.33	(5.43)	10.58	(5.43)	1.93	^†^
Anxiety (0–14)	6.95	(3.49)	6.34	(3.34)	7.55	(3.59)	1.33		7.89	(3.40)	6.13	(3.14)	1.96	^†^
Depression (0–14)	4.91	(3.23)	4.55	(3.55)	5.28	(2.90)	0.85		6.13	(3.41)	4.45	(3.29)	1.17	
Worry total (1–5)	2.42	(1.03)	2.24	(0.95)	2.60	(1.09)	1.38		2.70	(1.04)	2.19	(0.97)	1.97	^†^
Worry EGD procedure (1–5)	2.36	(1.16)	2.07	(1.02)	2.63	(1.24)	1.91	^†^	2.66	(1.22)	2.10	(1.06)	1.87	^†^
Worry EGD outcomes (1–5)	2.55	(1.20)	2.34	(1.07)	2.75	(1.30)	1.30		2.83	(1.16)	2.31	(1.20)	1.68	^†^
Worry general health (1–5)	2.43	(1.26)	2.47	(1.35)	2.40	(1.18)	0.20		2.67	(1.33)	2.23	(1.18)	1.32	

*^∗∗∗^p < 0.001; ^∗∗^p < 0.01; ^∗^p < 0.05; ^†^p < 0.05 (one-tailed test).*

Table 3 reports the reliability and validity statistics of latent and observed variables included in the predictive model outlined in Figure 1. The composite reliabilities were above the recommended threshold of 0.70 for all the latent variables in the model, ranging from 0.80 to 0.97. The AVE for the latent variables was much above the recommended standard of 0.50. The square roots of the AVE were also higher than the correlations of the latent variables with other latent variables in the model, thus supporting the discriminant validity criterion. Taken together, the analyses of the measurement model showed that the composite and indicator reliability, as well as the convergent and discriminant validity of the latent variables, were good.

**Table 3:** Reliability and validity of the latent variables.

	AVE	CR	1.	2.	3.	4.	5.	6.	7.	8.	9.	
1. Intolerance of uncertainty	0.94	0.97	0.97***									
2. Anxiety sensitivity	0.87	0.93	0.70***	0.93***								
3. Health distress	0.67	0.80	0.63***	0.60***	0.82***							
4. Worry	0.72	0.88	0.49***	0.45***	0.61***	0.85***						
5. Pain behavior	0.71	0.92	0.16***	0.23***	0.13***	0.16***	0.85***					
6. Self-report pain	0.69	0.90	0.20***	0.20***	0.21***	0.17***	0.56***	0.83***				
7. Pain catastrophizing	0.69	0.87	0.24***	0.36***	0.37***	0.43***	0.53***	0.62***	0.83***			
8. Female gender	1.00	1.00	0.05***	0.26***	0.18***	0.15***	0.08***	0.33***	0.35***	1.00		
9. Experienced patient	1.00	1.00	–0.15****	–0.15***	–0.25***	–0.24***	–0.11***	–0.16***	–0.24***	–0.16***	1.00	
10. Age	1.00	1.00	–0.07****	0.06***	–0.14***	–0.11***	–0.14***	–0.27***	–0.34***	0.00***	0.41***	1.00

***p 0.001; **p 0.01; *p 0.05 (one-tailed test based on 5000 bootstrap replications). Coefficients in the diagonal are the squared root of AVE. Coefficients below the diagonal are correlations among the latent variables in the model. Because gender, age, and endoscopy experience are observed variables, their CR and AVE are by definition equal to 1.00. .AVE, Average Variance Extracted; CR, Composite Reliability.

Intolerance of uncertainty, anxiety sensitivity, worry, and health distress were highly correlated. But the coefficients were not so large as to suggest an overlap of the constructs. Moreover, each of the psychological variables had specific relationships with the pain variables. IU and worry before EGD were associated with situational PC and self-reported pain after EGD. Health distress was significantly associated with situational PC, only. By contrast, anxiety sensitivity was correlated with all pain variables. The inspection of the correlation coefficients suggested that situational PC seemed to have a pivotal role, being significantly associated with all the psychological variables before EGD, and with the behavioral rating of and self-reported pain, during and after the procedure, respectively. As in previous descriptive analyses, female gender was significantly associated with anxiety sensitivity, while no previous endoscopy experience was related to greater health distress and worry. Older age was also negatively correlated with PC and self-reported pain.

**FIGURE 2:**
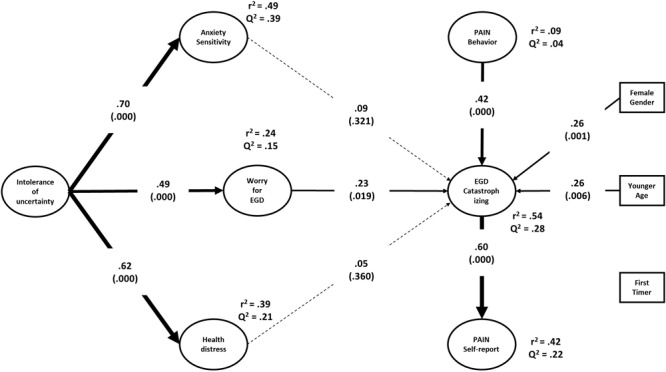
The estimated structural model.

Figure 2 shows the estimated structural model, including the path coefficients and *R*^2^ and *Q*^2^ statistics for the endogenous variables. The model accounted for 49, 39, and 24% (all *p*s < 0.001) of the variance in anxiety sensitivity, health distress, and EGD-related worry, respectively. IU was significantly associated with anxiety sensitivity (*f^2^* = 0.97), health distress (*f^2^* = 0.64), and worry (*f^2^* = 0.32). The model explained 54% (*p* < 0.001) and 42% (*p* < 0.001) of the variance in situational PC and self-reported pain, respectively. The prediction was not significant for pain behavior during EGD (*R*^2^= 0.09; *p* = 0.173). According to Hair et al. (2017), the effect sizes were large for situational PC and self-reported pain, and small-medium for pain behavior. The *Q*^2^-values were all positive, supporting the robustness of the model in terms of cross-validation.

Different from the correlations reported in Table 3, neither anxiety sensitivity nor health distress predicted any of the pain variables after controlling for each other and all the other variables in the model, including age, gender, and EGD experience. Only worry before EGD remained a significant predictor of situational PC (*f^2^* = 0.07). Regarding pain variables, the model showed that pain behavior during EGD predicted situation specific catastrophizing thoughts (*f^2^* = 0.34), which in turn were associated with self-reported pain after EGD (*f^2^* = 0.34). Female gender and patient’s age also predicted PC (*f^2^*s = 0.13 and 0.12, respectively). Unexpectedly, the patient’s experience with EGD did not predict any of the pain variables. In addition to testing specific path coefficients, the model allowed us to examine several indirect relationships among variables. Situational PC mediated the effect of pain behavior (Indirect effect = 0.25; 95% CI = [0.13, 0.38]; *p* = 0.001), gender (Indirect effect = 0.15; 95% CI = [0.05, 0.29]; *p* = 0.021), age (Indirect effect = -0.16; 95% CI = [-0.06, -0.28]; *p* = 0.008), worry (Indirect effect = 0.14; 95% CI = [0.03, 0.30]; *p* = 0.043) on self-reported pain. Moreover, worry mediated the effect of IU on situational PC (Indirect effect = 0.11; 95% CI = [0.03, 0.24]; *p* = 0.042).

Younger age and female gender, but not EGD experience, were associated with higher PC. Nevertheless, previous descriptive analyses had shown that first-timer patients tended to be more distressed before EGD and required higher doses to attain acceptable levels of sedation, too. Notwithstanding this, neither anxiety sensitivity nor health distress predicted situational PC. Neither, EGD experience seemed to play any role in the model. It is still entirely possible that the prediction of pain outcomes might depend on the interactive effects of psychological characteristics with age, gender, and EGD experience.

Using the available data, we tested specific hypotheses concerning the role of EGD experience (as well as those of age and gender) as factors that might alter the average level of PC as a function of anxiety sensitivity, worry, and health distress. As one can see from Table 4, EGD experience and female gender moderated the prediction of PC by anxiety sensitivity and health distress. Patient’s age was also a significant moderating factor in all of the predictive relationships mentioned above. By contrast, no moderation effects were detected for pain-behavior and self-reported pain as dependent variables, which remained not such greatly affected from the psychological status of the patients before EGD (except the indirect effect of worry on self-reported pain).

**Table 4:** Tests of moderation effects.

Effect	Beta	LLCI	ULCI	*t*-value	p	Effect	Beta	LLCI	ULCI	*t*-value	p	Effect	Beta	LLCI	ULCI	*t*-value	p
AS × EXP – > PBR	–0.18	–0.42	0.07	1.15		WO × EXP – > PBR	0.02	–0.19	0.27	0.14		HD × EXP – > PBR	–0.03	–0.30	0.23	0.21	
AS × EXP – > PCS	–0.29	–0.48	–0.10	2.43	^∗∗^	WO × EXP – > PCS	–0.04	–0.04	0.12	0.22		HD × EXP – > PCS	–0.26	–0.45	–0.07	2.25	^∗^
AS × EXP – > PSR	–0.03	–0.28	0.17	0.22		WO × EXP – > PSR	0.16	–0.02	0.38	1.29		HD × EXP – > PSR	0.00	–0.24	0.22	0.01	
AS × FEM – > PBR	–0.07	–0.36	0.19	0.40		WO × FEM – > PBR	0.03	–0.22	0.27	0.20		HD × FEM – > PBR	0.08	–0.23	0.36	0.44	
AS × FEM – > PCS	0.22	0.05	0.41	1.98	^∗^	WO × FEM – > PCS	0.07	–0.13	0.23	0.67		HD × FEM – > PCS	0.22	0.05	0.41	1.98	^∗^
AS × FEM – > PSR	0.10	–0.15	0.33	0.70		WO × FEM – > PSR	0.03	–0.20	0.27	0.23		HD × FEM – > PSR	0.14	–0.06	0.38	0.99	
AS × AGE – > PBR	–0.03	–0.23	0.21	0.20		WO × AGE – > PBR	–0.08	–0.30	0.19	0.54		HD × AGE – > PBR	0.00	–0.24	0.28	0.00	
AS × AGE – > PCS	–0.41	–0.62	–0.26	3.76	^∗∗∗^	WO × AGE – > PCS	–0.37	–0.57	–0.15	2.77	^∗∗^	HD × AGE – > PCS	–0.29	–0.45	–0.07	2.31	^∗^
AS × AGE– > PSR	–0.11	–0.30	0.08	0.91		WO × AGE – > PSR	–0.04	–0.25	0.17	0.29		HD × AGE – > PSR	–0.18	–0.39	0.04	1.26	

*^∗∗∗^p < 0.001; ^∗∗^p < 0.01; ^∗^p < 0.05 (one-tailed test based on 5000 bootstrap replications). AS, anxiety sensitivity; EXP, previous EGD experience; FEM, female gender; AGE, patient’s age; WO, worry; HD, health distress; PBR, pain, behavioral rating; PSR, pain. self-report; PCS, pain catastrophizing. LLCI, 95% Lower Limit Confidence Interval; ULCI, 95% Upper Limit Confidence Interval.*

**FIGURE 3:**
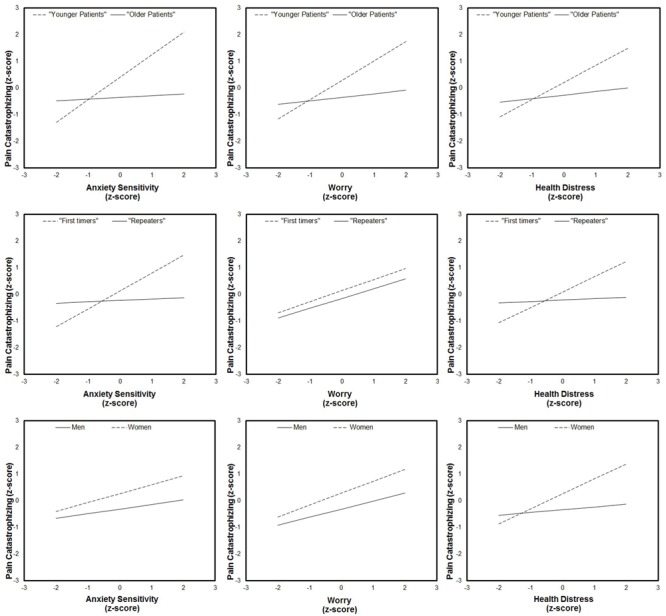
Simple slope analysis.

Because a moderation effect could be very informative about “under which circumstances” or “for whom,” an independent variable is more (or less) strongly associated with the outcomes, we examined the simple slopes for situational PC on the three independent variables for men and women, first-timers and repeaters, and younger and older patients. As one can see from Figure 3, PC significantly increased with anxiety sensitivity, worry, and health distress, but only for younger patients. Likewise, anxiety sensitivity and health distress significantly predicted situational PC thoughts, but only for first-time patients and women.

## Discussion

Being focused on anxiety symptoms, previous research has overlooked the role of their cognitive antecedents, which lead patients to experience overwhelming anxiety before EGD, misinterpret uncomfortable physical sensations, and increase the risks and the costs associated with over-sedation (Jones et al., 2004; Essink-Bot et al., 2007; Pontone et al., 2015; Behrouzian et al., 2017). Using a two-stage prospective design, the present study adds to the extant literature showing that IU, anxiety sensitivity, health distress, and worry are associated with subsequent clinical outcomes in the pain domain. Each of the four cognitive variables assessed before endoscopy had a specific predictive relationship with PC, self-reported pain, and behavioral pain ratings assessed after and during EGD, respectively.

First of all, our study showed that IU assessed before endoscopy was associated with situational PC and self-reported pain after endoscopy. Paralleling previous research on anxiety disorders (Dugas et al., 2005; Shihata et al., 2016), our multivariate analysis showed that IU prompted specific EGD-related worries, which in turn were predictive of pain-catastrophizing and pain. Thus, patients high on IU reacted to the possibility of complications or unfavorable test results, worrying about the negative events that might happen during or after the procedure. In the endoscopy situation, worrying is maladaptive in that it cannot prevent any of the feared negative events from occurring (De Bruin et al., 2007; Newman et al., 2013). Rather, worrying about the impending procedure led patients to ruminate and catastrophize about unpleasant physical sensations, which in turn increased the likelihood of tolerating EGD less well.

A second major finding of the study was that situational PC had a pivotal role in the chain of events that lead to low tolerability. Not only it mediated the effect of worry, but also female gender and younger age were both associated with self-reported pain through increased catastrophizing thoughts. Foreshadowing the discussion of the clinical implications, it looks like that PC thoughts during endoscopy were likely responsible for low tolerability for these specific groups of patients (Davies and Roy, 2013; Lee et al., 2014; Gürbulak et al., 2018; Sayilan and Oztekin, 2018). Our findings add to extant literature showing that situational PC has sound relationships with the patient’s perceived pain, as also shown by studies of patients undergoing other invasive medical procedures or surgery (Strulov et al., 2007; Campbell et al., 2010; Khan et al., 2011; Pinto et al., 2012; Grosen et al., 2016).

Patient’s health distress and anxiety sensitivity were significantly associated with situational PC in bivariate analyses. Also, anxiety sensitivity was correlated with evident pain behaviors during endoscopy. However, subsequent analyses showed that these correlations did not yield significant regression paths in multivariate analyses. Different from worry, the prediction of PC by health distress and anxiety sensitivity was moderated by the other known risk factors for low tolerability. In particular, the two anxiety-related variables affected subsequent catastrophizing thoughts only for women, younger patients, and those not accustomed to the procedure. These findings corroborate previous research aimed at identifying which patients tolerate digestive endoscopy less well (Davies and Roy, 2013; Lee et al., 2014; Gürbulak et al., 2018; Sayilan and Oztekin, 2018) and suggested that addressing health distress and anxiety sensitivity is of utmost importance to make these groups of patients more compliant and easy to examine (Hazeldine et al., 2010; Bal et al., 2012).

The lack of information about what might happen during a medical procedure is an important distressing factor, able to activate cognitive distortions in the appraisal of threat (Dugas et al., 2005; Shihata et al., 2016). In keeping with this view, we hypothesized first-timers to be more distressed and worried before endoscopy, as also noticed in a previous study (Davies and Roy, 2013). This hypothesis was supported in the present study, too. Noteworthy, first-timers also needed higher Midazolam doses than more experienced patients. Because first-timers did not differ from repeaters in the average BMI, it seems likely that the medical personnel – blinded to the preceding psychological assessment – judged first-timers as ostensibly more agitated before the procedure and opted for administering higher sedative doses to. After all, previous research had already shown that the more apprehensive patients are more difficult to sedate (e.g., Lee et al., 2004; Bal et al., 2012; Gürbulak et al., 2018), and our findings are consistent with this view.

Although EGD is typically well tolerated under conscious sedation, our study showed that more than half of the patients were above the cut-off value for probable pain using a behavioral rating scale. Because most patients attained an adequate level of sedation, it is worth asking whether the patients coped with a “real pain” or just misjudged unpleasant physical sensations as pain (Ocañez et al., 2010). Previous research has shown that psychological preparation for EGD is more effective if the patient is acquainted with the physical sensations that he/she will experience (Maguire et al., 2004; García Sierra et al., 2013; Kowsalya et al., 2015; Behrouzian et al., 2017; Liu et al., 2018; Ghonaem and Ibrahim, 2019). Merely providing information about the procedure can even be counterproductive for some patients (Bytzer et al., 2007).

Before concluding it is worth acknowledging some limitations of the present study. First, the sample size was relatively small. Although the number of patients was adequate for performing non-parametric analyses, it precluded us from controlling other qualitative variables potentially influencing pre-procedural anxiety, like motives for a referral to the endoscopy center or final diagnosis after endoscopy. Second, PC and self-reported pain were both assessed after the procedure. This might threaten the directional interpretation of our findings concerning the prediction of self-reported pain based on a concurrent assessment of PC. Last, we recruited patients at only one university clinic, and only one endoscopist performed all exams. Thus, the findings of the present study should be cautiously interpreted regarding their generalizability to smaller clinics, private diagnostic wards, or less skilled operators.

Its limitations notwithstanding, our study is the first to address the interplay of IU, anxiety sensitivity and PC in relation to EGD tolerability. Moreover, our findings may enable identification of potential avenues for psychological intervention in the endoscopy setting. While relaxing music or aromatic care have been suggested to defuse procedural anxiety (e.g., Rudin et al., 2007; Hu et al., 2014), these interventions miss the psychological elements highlighted in the present study. Indeed, procedural anxiety is likely to evolve into painful sensations when the patient – especially if young, female, or first-timer – negatively appraises the unusual bodily sensations and the mental events associated with EGD. Accordingly, part of patient preparation should be focused on replacing worries and catastrophic thoughts with more positive appraisals of the medical examination and its results addressing negative beliefs and anticipated emotions. This is typically done in chronic pain using cognitive behavioral therapy that requires weeks before producing appreciable results. A major constraint for psychological interventions in the endoscopy suite is the time needed to produce effective reappraisals. Some studies, however, sound promising. For instance, EGD patients who received psychological preparation 2–3 h prior the endoscopy significantly reduced procedural anxiety through information about endoscopy, cognitive preparation (e.g., positively reframing the situation), and behavioral interventions (e.g., breathing exercising, swallowing training) (Behrouzian et al., 2017). Likewise, a 12 min preparation reduced patient’s distress during endoscopy using information about the sensations and sequence of events associated with EGD and behavioral training based on breathing exercises, swallowing technique, and a tongue depressor task (Maguire et al., 2004).

Although promising, none of the studies mentioned above has targeted IU, anxiety sensitivity and PC, nor considered pain as the primary outcome. An avenue for future research could be designing interventions in which patient training is aimed to replace negative beliefs with positive coping self–statements (e.g., “I can handle this, just relax”), that is a recommended strategy to cope with acute pain through psychological preparation (Bruehl and Chung, 2003). Interventions based on a clinical approach to case studies (Langher et al., 2017) could also shed light on whether reducing maladaptive beliefs in first-timers, women, and younger patients lead to corresponding improvements in EGD tolerability as predicted by our model.

## Data Availability

The datasets generated for this study are available on request to the corresponding author.

## Ethics Statement

All procedures performed in studies involving human participants were in accordance with the ethical standards of the institutional and/or national research committee and with the 1964 Helsinki declaration and its later amendments or comparable ethical standards. Informed consent was obtained from all individual participants included in the study.

## Author Contributions

This manuscript updates and extends a preliminary research report presented at the Digestive Disease Week 2018. The final version of this manuscript was written by ML, MT, and SP, who contributed equally to the theoretical and empirical aspects of the study. RP contributed to the collection and analysis of medical data and wrote a preliminary version of this manuscript. GL and AF collected and analyzed the psychological data. CP and MR collected and analyzed the medical data.

## Conflict of Interest Statement

The authors declare that the research was conducted in the absence of any commercial or financial relationships that could be construed as a potential conflict of interest. The handling Editor declared a past co-authorship with one of the authors MT.
